# Factors associated with in-hospital mortality in adult sepsis with *Escherichia coli* infection

**DOI:** 10.1186/s12879-022-07201-z

**Published:** 2022-02-28

**Authors:** Kun Song, Cuirong Guo, Zhao Zeng, Changluo Li, Ning Ding

**Affiliations:** Department of Emergency Medicine, The Affiliated Changsha Central Hospital, Hengyang Medical School, University of South China, NO.161 Shaoshan South Road, Changsha, 410004 Hunan China

**Keywords:** RDW, HCT, Sepsis, *E. coli*, Prognosis

## Abstract

**Background:**

*Escherichia coli* (*E. coli*) is an important pathogen in sepsis. This study aimed to explore the factors which were associated with in-hospital mortality in adult sepsis with *E. coli* infection based on a public database.

**Methods:**

All sepsis patients with *E. coli* infection in MIMIC-III were included in this study. Clinical characteristics between the survivor and non-survivor groups were analyzed. Factors associated with in-hospital mortality were identified by multivariate logistic regression.

**Results:**

A total of 199 patients were eventually included and divided into two groups: a survivor group (n = 167) and a non-survivor group (n = 32). RDW and HCT were identified as the factors with clinical outcomes. The area under the ROC curve (AUC) were 0.633 and 0.579, respectively. When combined RDW and HCT for predicting in-hospital mortality, the AUC was 0.772, which was significantly superior to SOFA and APACHEII scores.

**Conclusion:**

RDW and HCT were identified as factors associated with in-hospital mortality in adult sepsis patients with *E. coli* infection. Our findings will be of help in early and effective evaluation of clinical outcomes in those patients.

## Introduction

Sepsis has been defined as a dysregulated host immune response to infections, leading to a life-threatening organ dysfunction [1]. *Escherichia coli* (*E. coli*) as one major kind of gram-negative bacilli may cause intra-abdominal infections, urinary tract infections, and sepsis [2]. An early-onset neonatal sepsis research with 235 cases showed that the most frequent pathogen was *E. coli* (86 [36.6%]) with higher incidence of mortality [3]. In China, a recent study clarified that *E. coli* infection accounted for nearly 30% in neonatal sepsis with more than a 10% death rate [4]. The immature immune systems of neonates may lead to a higher mortality in *E. coli* infection. Hence, early identification of those sepsis patients with poor prognosis was significant.

However, for *E. coli* infection, most previous studies focused on neonatal sepsis and few studies have been done for investigating the clinical characteristics of adult patients. Moreover, little has been known about the predictive values of different laboratory variables in adult sepsis with *E. coli* infection. Therefore, in our study, we aimed to explore the factors which were associated with in-hospital mortality in adult sepsis with *E. coli* infection based on a public database.

## Methods

### Patients

All sepsis patients with *E. coli* infection in MIMIC-III were included in this study. MIMIC-III database as an US-based critical care public database includes data linked with 53,423 adult patients (aged 16 years or above) from 2001 to 2012 and 7870 neonates from 2001 to 2008 admitted to a intensive care unit (ICU) [5]. Data including vital signs, medications, laboratory measurements, observations and notes charted by care providers, fluid balance, procedure codes, diagnostic codes, imaging reports, hospital length of stay and survival data were comprehensively recorded. The following tables in MIMIC III dataset were utilized in our study: ADMISSIONS, CHARTEVENTS, D_ICD DIAGNOSIS, D_ITEMS, D_LABIEVENTS, DIAGNOSIS_ICD, ICUSTAYS, LABEVENTS, NOTEEVENTS, PATIENTS, INPUTEVENTS_CV, INPUTEVENTS _MV and OUTPUTEVENTS [5].

### Study population

All patients with a diagnosis relevant to sepsis with *E. coli* infection in the database were initially screened. The diagnosis of sepsis with *E. coli* infection in the database was confirmed by the lab findings when the pathogen culture in blood was positive in *E. coli*. Only the data of each patient in the first admission were utilized in this study. Exclusion criteria included as follows: patients with missing > 5% individual data and age less than 18.

### Data extraction

Data extraction was performed by using structure query language (SQL). The data of demographic characteristics, clinical variables, laboratory variables and scoring systems were extracted for further analysis. The baseline characteristics used were those recorded within 24 h after admission. When one variable was recorded at a different time compared to the initial 24 h, the first one was enrolled in the study. Demographic characteristics included age, gender, marital status, ethnicity, ICU department, admission type, and comorbidities (renal disease, coronary artery disease (CAD), diabetes, hypertension). Clinical and laboratory variables included systolic blood pressure (SBP), diastolic blood pressure (DBP), heart rate (HR), respiratory rate (RR), white blood cells (WBC), neutrophils, lymphocytes, basophils, platelet (PLT), red cell volume distribution width (RDW), hematocrit (HCT), glucose, prothrombin time (PT), thrombin time (TT), albumin, alanine aminotransferase (ALT), aspartate aminotransferase (AST), mean corpuscular volume (MCV), total bilirubin, creatinine, lactate, total calcium and anion gap. Clinical outcomes including length of stay (LOS) in ICU and in-hospital mortality and scoring systems including sequential organ failure assessment (SOFA) and acute physiology and chronic health evaluation (APACHEII) were also extracted.

### Statistical analysis

Characteristics are expressed as mean ± standard deviation or median (IQR) for continuous variables and a percentage or frequency for categorical variables. Continuous variables were compared using Student’s t-test (normal distribution) or Mann–Whitney U-test (Skewed distribution), and categorical variables were compared using Fisher’s exact test or Chi-square analysis. Stepwise logistic regression for variables selection in multivariable logistic regression was performed. Variables with P < 0.2 which were compared between the survivor and non-survivor groups were further enrolled in multivariable logistic regression. Then, factors associated with in-hospital mortality was identified by multivariate logistic regression. Finally, the receiver-operator characteristic (ROC) analysis of different factors for predicting in-hospital mortality were performed. The cut-off values of variables were confirmed by the Youden Index (sensitivity + specificity-1). The value of each variable with the maximum Youden Index was the cut-off value.

SPSS software (version 26) was implemented for statistical analysis. Two-sided P values < 0.05 were considered statistically significant.

## Results

### General characteristics of the patients

**Fig. 1:**
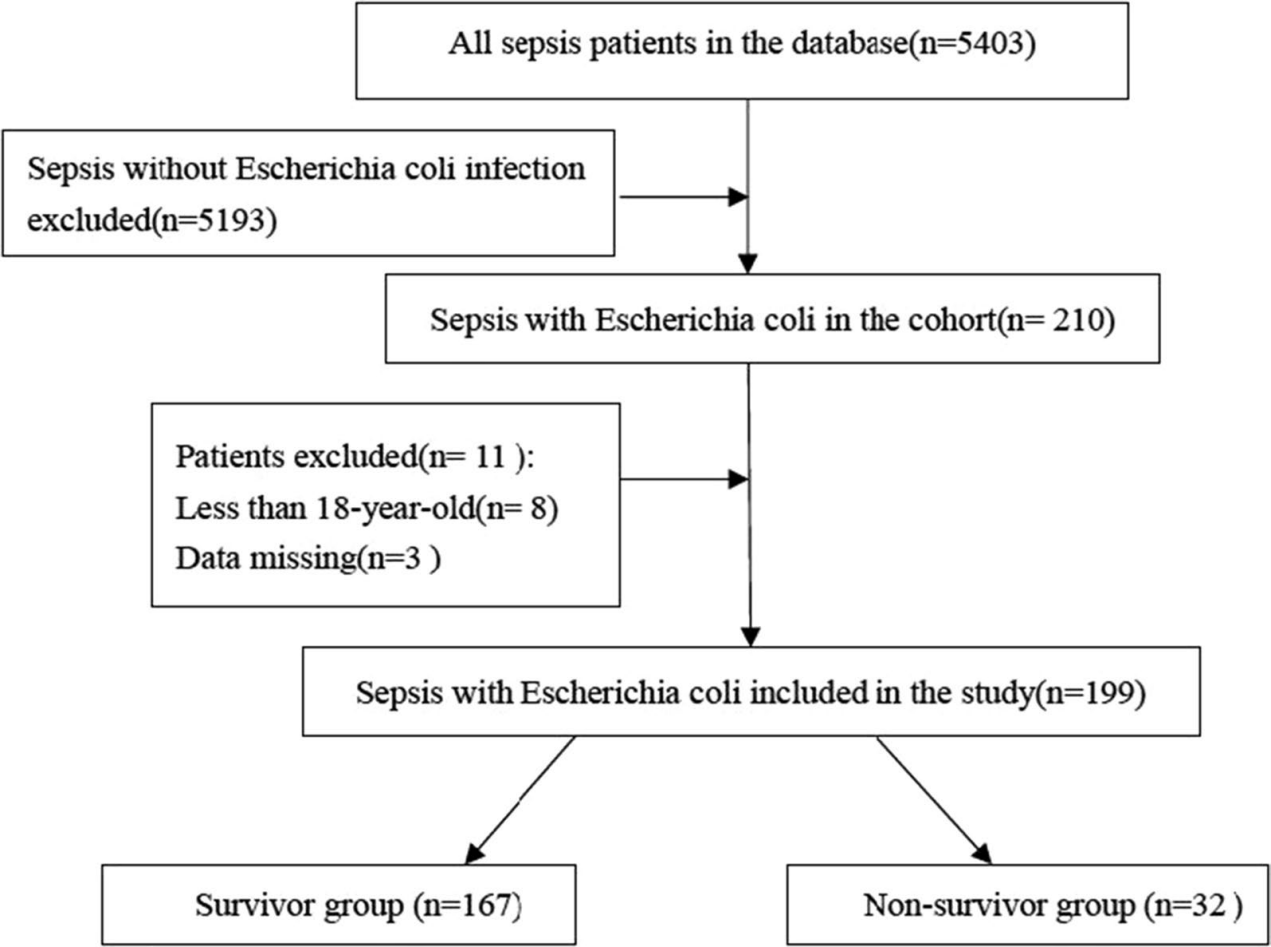
Flow chart for patients enrollment and study design

At first, 5403 sepsis patients were included. Then, based on the infection of different pathogens, 210 sepsis patients with *E. coli* infection were enrolled in this research. According to the exclusion criteria, 11 patients were excluded and a total of 199 patients were included and divided into a survivor group (n = 167) and a non-survivor group (n = 32) (Fig. 1). General characteristics of the cohort were elucidated in Table 1. The median age was 69.52 and males accounted for 45.22% in total. Most of the patients were hospitalized in MICU (83.42%) and emergency admission was the most common admission type (96.98%). The top four comorbidities were as follows: hypertension (45.73%), CAD (18.09%), diabetes (4.52%) and renal disease (4.02%). The median scores of APACHEII and SOFA were 14 and 3, respectively.

**Table 1:** General characteristics of the patients

Variables	
Number of patients (n)	199
Age (years)	69.52 (61.01–82.35)
Gender (n, %)	
Male	90 (45.22%)
Female	109 (54.78%)
Marital status (n, %)	
Divorced	14 (7.03%)
Married	83 (41.72%)
Single	58 (29.14%)
Widow	33 (16.58%)
Others	11 (5.53%)
Ethnicity (n, %)	
Asian	14 (7.04%)
White	142 (71.36%)
Black/American	14 (7.03%)
Hispanic/Latino	4 (2.01%)
Others	25 (12.56%)
Department (n, %)	
CCU	12 (6.03%)
MICU	166 (83.42%)
SICU	12 (6.03%)
TICU	5 (2.51%)
CSRU	4 (2.01%)
Admission type (n, %)	
Elective	4 (2.01%)
Urgent	2 (1.01%)
Emergency	193 (96.98%)
Comorbidities (n, %)	
Renal disease	8 (4.02%)
CAD	36 (18.09%)
Diabetes	9 (4.52%)
Hypertension	91 (45.73%)
Scoring system	
APACHEII	14 (12–17)
SOFA	3 (1–4)
Clinical outcomes	
LOS in ICU (days)	3.70 (1.96–8.97)
LOS in hospital (days)	8 (5–17)
In-hospital mortality (n, %)	32 (16.08%)

*SOFA* sequential organ failure assessment, *APACHE* acute physiology and chronic health evaluation, *LOS* length of stay, *ICU* intensive care unit, *CAD* coronary artery disease

The median days of LOS in ICU and in hospital were 3.7 and 8, respectively. In-hospital mortality was 16.08%.

### Comparison of variables between survivor and non-survivor groups

Different variables in survivor and non-survivor groups were compared and analyzed in Table 2. The median age of the survivor and non-survivor groups were 69.96 and 68.32 (P = 0.573). Gender showed no significant difference (P = 0.171). In vital signs, no significant differences showed in DBP (P = 0.414), SBP (P = 0.138) and RR (P = 0.068), while HR was significantly higher in the non-survivor group (P = 0.043). Comparison of comorbidities including renal disease (P = 0.778), CAD (P = 0.916), hypertension (P = 0.149) and diabetes (P = 0.806) demonstrated no significant differences between the two groups. In laboratory characteristics, PLT (P = 0.551), AST (P = 0.863), MCV (P = 0.278) glucose (P = 0.475), ALT (P = 0.789), TT (P = 0.733), hematocrit (P = 0.060), PT (P = 0.935), anion gap (P = 0.273), lymphocytes (P = 0.590), WBC (P = 0.479), lactate (P = 0.078), albumin (P = 0.369), creatinine (P = 0.728), total bilirubin (P = 0.176) and calcium (P = 0.854) didn’t have any significant differences between the two groups. Neutrophils (P = 0.015), RDW (P = 0.026) and basophils (P = 0.021) showed significant differences. There was no significant difference in the scores of APACHEII (P = 0.585) and SOFA (P = 0.357). In the non-survivor group, the days of LOS in ICU (P < 0.001) and hospital (P = 0.032) were longer.

**Table 2:** Comparison of variables between survivor and non-survivor groups

Variables	Survivor (n = 167)	Non-survivor (n = 32)	P-value
Age (years)	69.96 ± 14.80	68.32 ± 16.04	0.573
Gender (n, %)			
Male	72 (43.11%)	18 (56.25%)	0.171
Female	95 (56.89%)	14 (43.75%)	
Vital signs			
DBP (mmHg)	59.00 (48.50, 71.50)	63.00 (52.00, 69.00)	0.414
SBP (mmHg)	110.83 ± 24.586	117.84 ± 23.489	0.138
HR (beats/min)	97.46 ± 19.739	105.22 ± 19.511	0.043
RR (beats/min)	20.00 (16.00, 25.00)	22.50 (20, 28.75)	0.068
Comorbidities (n,%)			
Renal disease	7 (4.19%)	1 (3.12%)	0.778
CAD	30 (17.96%)	6 (18.75%)	0.916
Diabetes	6 (3.59%)	3 (9.37%)	0.149
Hypertension	77 (46.11%)	14 (43.75%)	0.806
Laboratory characteristics			
PLT (*10^9^/L)	189.00 (132.50, 295.00)	198.00 (125.00, 310.00)	0.551
AST (IU/L)	47.00 (25.50, 90.00)	48.00 (26.00, 92.00)	0.863
MCV (fL)	91.00 (88.00, 96.00)	91 (87.50, 95.00)	0.278
Glucose (mg/dL)	121.00 (103.50, 163.50)	138.00 (116.00, 166.00)	0.475
ALT (IU/L)	33.00 (19.50, 85.00)	38.00 (17.00, 54.00)	0.789
Neutrophils (%)	78.50 (66.50, 82.50)	82.00 (75.00, 89.90)	0.015
TT (s)	30.40 (26.35, 35.40)	32.70 (27.70, 39.50)	0.733
HCT (%)	34.16 ± 5.41	36.43 ± 8.28	0.060
PT (s)	14.40 (13.15, 17.30)	14.90 (12.80, 19.40)	0.935
Anion Gap (mmol/L)	17.00 (13.00, 20.00)	17.00 (13.00, 21.00)	0.273
RDW (%)	14.87 ± 1.85	15.70 ± 2.18	0.026
Lymphocytes (%)	6.00 (2.95, 12.00)	8.00 (5.00, 15.00)	0.590
WBC (*10^9^/L)	12.20 (6.30, 19.75)	13.50 (3.70, 18.30)	0.479
Lactate (mmol/L)	2.40 (1.45, 3.45)	2.90 (1.60, 4.50)	0.078
Albumin (g/dL)	3.00 (2.50, 3.50)	2.90 (2.50, 3.40)	0.369
Creatinine (mg/dL)	1.30 (1.00, 2.150	1.40 (0.90, 2.50)	0.728
Total bilirubin (mg/dL)	0.80 (0.40, 1.60)	1.10 (0.50, 3.70)	0.176
Total calcium (mg/dL)	8.02 ± 0.964	7.99 ± 1.383	0.854
Basophils (%)	0.00 (0.00, 0.20)	0.00 (0.00, 0.10)	0.021
Scoring system (IQR)			
APACHEII	14.00 (12.00, 18.00)	15.50 (10.50, 17.00)	0.585
SOFA	2.00 (1.00, 4.00)	3.00 (2.00, 4.75)	0.357
Clinical outcomes (days)			
LOS in ICU	3.22 (1.87, 7.82)	11.01 (4.66, 17.75)	< 0.001
LOS in hospital	8.00 (5.00, 15.00)	13.50 (6.25, 35.50)	0.032

*SBP* systolic blood pressure, *DBP* diastolic blood pressure, *HR* heart rate, *RR* respiratory rate, *CAD* coronary artery disease, *WBC* white blood cells, *PLT* platelet, *RDW* red cell volume distribution width, *PT* prothrombin time, *TT* thrombin time, *ALT* alanine aminotransferase, *AST* aspartate aminotransferase, *HCT* hematocrit, *MCV* mean corpuscular volume, *SOFA* sequential organ failure assessment, *APACHE* acute physiology and chronic health evaluation, *LOS* length of stay, *ICU* intensive care unit, *IQR* interquartile ranges

### Factors associated with in-hospital mortality in multivariable analysis

Variables including gender (male), SBP, HR, RR, diabetes, neutrophils, HCT, RDW, lactate, total bilirubin and basophils were enrolled in multivariable analysis (Table 3). Two factors associated with in-hospital mortality were identified: HCT (P = 0.007, Odds Ratio (OR) = 1.116, 95%CI = 1.030–1.209) and RDW (P = 0.002, OR = 1.435, 95%CI = 1.140–1.806).

**Table 3:** Factors associated with in-hospital mortality in multivariable analysis

Variables	B	SE	Wald	P value	OR	95% CI for OR	
						Lower	Upper
Male	0.406	0.464	0.764	0.382	1.500	0.604	3.727
SBP	0.008	0.010	0.654	0.419	1.008	0.989	1.027
HR	0.004	0.013	0.087	0.768	1.004	0.978	1.031
RR	0.060	0.040	2.249	0.134	1.062	0.982	1.149
Diabetes	1.527	0.942	2.625	0.105	4.603	0.726	29.174
Neutrophils	− 0.023	0.011	4.017	0.055	0.978	0.956	1.018
HCT	0.110	0.041	7.227	**0.007**	1.116	1.030	1.209
RDW	0.361	0.117	9.459	**0.002**	1.435	1.140	1.806
Lactate	0.024	0.124	0.036	0.849	1.024	0.804	1.305
Total Bilirubin	0.059	0.052	1.321	0.250	1.061	0.959	1.174
Basophils	− 0.672	0.814	0.682	0.409	0.511	0.104	2.517

*CI* confidential interval, *OR* odds ratio, *RDW* red cell volume distribution width, *SBP* systolic blood pressure, *HR* heart rate, *RR* respiratory rate, *HCT* hematocrit

### Predictive performances of factors and scoring systems

**Fig. 2:**
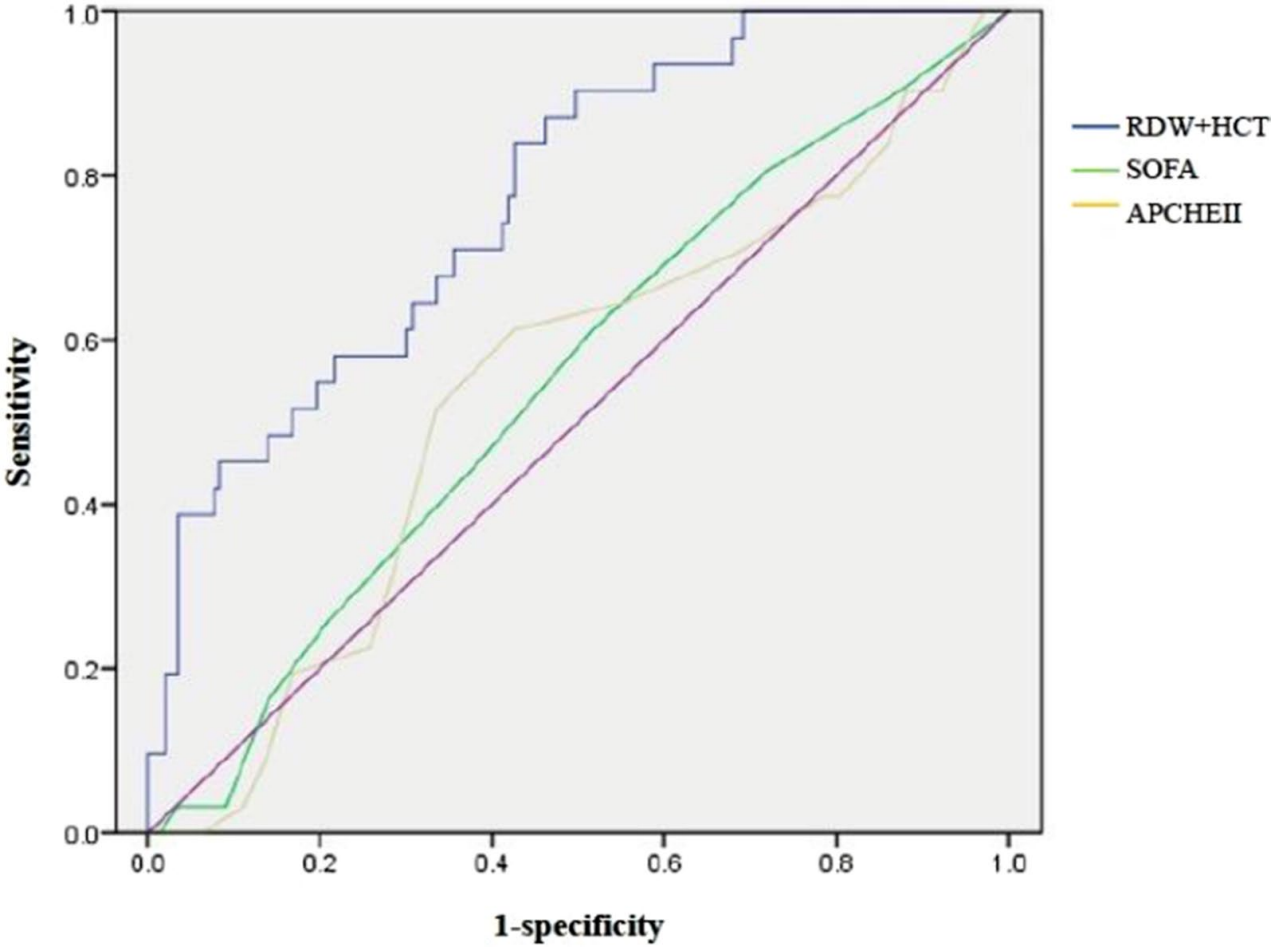
ROCs of different models. *SOFA* sequential organ failure assessment, *APACHE* acute physiology and chronic health evaluation, *RDW* red cell volume distribution width, *HCT* hematocrit

In Table 4 and Fig. 2, different predictive performances of HCT, RDW and scoring systems including SOFA and APAHEII were demonstrated. The cut-off values of RDW and HCT were 15.45% and 38.4%, respectively. The area under the ROC curve (AUC) of RDW and HCT were 0.633 and 0.579, respectively. When combined RDW and HCT for predicting in-hospital mortality, the AUC was 0.772, which was significantly superior to SOFA and APACHEII scores.

**Table 4:** Predictive performances of RDW, HCT and scoring systems

Variables	AUC	95%CI	Cut-off value	Sensitivity (95%CI)	Specificity (95%CI)
RDW (%)	0.633	0.518–0.748	15.45	0.625 (0.437–0.783)	0.686 (0.611–0.756)
HCT (%)	0.579	0.451–0.706	38.4	0.438 (0.268–0.621)	0.801 (0.732–0.858)
RDW + HCT	0.772	0.687–0.858	–	–	–
SOFA	0.550	0.441–0.658	3	0.613 (0.407–0.757)	0.487 (0.425–0.580)
APACHEII	0.539	0.427–0.651	14	0.613 (0.408–0.758)	0.573 (0.502–0.655)

*AUC* area under the ROC curve, *CI* confidential interval, *SOFA* sequential organ failure assessment, *APACHE* acute physiology and chronic health evaluation, *RDW* red cell volume distribution width, *HCT* hematocrit

## Discussion

In our retrospective study, RDW and HCT were identified as factors associated with in-hospital mortality in adult sepsis patients with *E. coli* infection. To the best of our knowledge, this was the first study to explore the association of the factors with clinical prognosis in adult sepsis with *E. coli* infection based on MIMIC-III public database.

RDW as a parameter which could measure the range of variation of red blood cell size has been proved to be a common and inexpensive biomarker in critical illness [6]. Elevated RDW levels implicated higher variation in size, which has been usually applied for differentiation in anemia due to nutritional deficiency [7].

There is an accumulation of studies that have explored the association between RDW and clinical outcomes in sepsis. Recently, two modified and simple scores both including RDW have been proved to be useful tools for predicting short-term outcomes in sepsis or septic shock [8, 9]. One study focusing on neonatal sepsis elucidated that RDW to platelet ratio as a useful systemic inflammatory marker could be an indicator for sepsis occurrence in early stage [10]. In adult sepsis patients, the combination of three parameters including RDW, platelet distribution width and the neutrophil‑lymphocyte count ratio which were easily acquired from whole blood cell count analysis had a good diagnostic performance [11]. A nomogram including RDW provided a relatively accurate prediction for the early identification of septic patients at high risk of mortality in the emergency department [12]. One meta-analysis including 17,961 sepsis patients from 11 studies demonstrated that RDW was a significantly useful predictor of mortality in sepsis and patients with elevated RDW were more likely to have higher mortality [13].

Studies on RDW in different cohorts had different cut-off values. In a study with a total of 103 patients with community-acquired intra-abdominal sepsis, RDW ≥ 16 had an AUC of 0.867 for predicting in-hospital mortality [14]. Another study with 1046 patients concluded that for 30-day mortality and early clinical deterioration, an optimal cut-off value of RDW were 12.95 and 14.48, respectively [15]. One recent study on sepsis patients demonstrated that Youden Index was maximum (37%) at RDW value 14.75, which was good at predicting mortality within 28-days of emergency admission [16]. In our study, the best threshold value of RDW for predicting in-hospital mortality was 15.45.

The underlying mechanisms as to why increased RDW was associated with adverse prognosis in sepsis remained largely unknown, but several explanations have been illuminated in some studies. First, elevated inflammatory markers due to systemic inflammation response in sepsis may affect the erythrocytes maturation and lead to the migration of reticulocytes into the peripheral circulation, thereby resulting in RDW being elevated [17]. Second, reactive erythropoiesis was stimulated under oxidative stress which was one of the pathophysiologic entities of sepsis. Then, large immature red cells with poor oxygen-binding capacity were released, causing an increase in the RDW [18]. Third, sepsis can interrupt the iron steady state, trigger bone marrow suppression, and downregulate the expression of the erythropoietin receptor, which all contribute and cause more production of ineffective red blood cell and RDW increased [19].

In our research, HCT was another factor which was associated with in-hospital mortality in sepsis. One recent study based on machine learning for early detection of late-onset neonatal sepsis showed that HCT was one of top three predictive variables [20]. Another study in Brazil found that as a predictor of mortality risk in the sepsis, the level of HCT decreased with worse outcomes [21]. However, a positive relationship between HCT and mortality was found in our study, which was not consistent with some previous studies [22, 23]. The differences could be partly explained by two reasons. First, sepsis patients with poor outcomes were more likely to be suffering from hypovolemia due to increased capillary permeability [24], which resulted in higher levels of HCT. Second, the general characteristics of sepsis patients in different studies were not the same.

Limitations should also be clarified in our study. First, the study was on the basis of a publicly single-center database in US. While applying to other nations, concerns regarding the generalizability of the conclusions and the confounding bias caused by the missing data should be considered. Second, the new definition of Sepsis-3 was not included in this study because the patients in MIMIC-III were enrolled before 2012, which may lead to some limitations in applying our results. Third, RDW is always related to the underlying condition, especially chronic anemia, while anemia is one of the most common complications in patients with sepsis in the ICU [25]. Sepsis-related anemia can be caused by some factors including fluid loading-related hemodilution, iatrogenic blood loss, and inflammation-associated abnormalities in erythropoiesis [26, 27]. Due to lack of some data in MIMIC-III, the anemia which involved past medical history or caused by sepsis couldn’t be defined clearly. Further research should be done for exploring the differences between sepsis with anemia and without anemia in order to validate our results. Fourth, samples in our study were relatively small and subgroups were not divided for further analysis. Due to lack of some data in MIMIC-III, not all the variables which may affect the association between RDW and prognosis were enrolled. Hence, more samples with more variables and multiple centers should be explored for validating our results.

## Conclusion

RDW and HCT were identified as factors associated with in-hospital mortality in adult sepsis with *E. coli* infection. Our findings will be of help in early and effective evaluation of clinical outcomes in those patients. Therefore, the measurements of RDW and HCT should be considered for prognostic assessment of adult sepsis with *E. coli* infection.

## Data Availability

The data that support the findings of this study are available from the Massachusetts Institute of Technology (MIT) and Beth Israel Deaconess Medical Center (BIDMC) but restrictions apply to the availability of these data, which were used under license for the current study, and so are not publicly available. Data are however available from the authors upon reasonable request and with permission of the Massachusetts Institute of Technology (MIT) and Beth Israel Deaconess Medical Center (BIDMC).

## Abbreviations

*E. coli*: *Escherichia coli*
SOFA: Sequential organ failure assessment
APACHE: Acute physiology and chronic health evaluation
LOS: Length of stay
ICU: Intensive care unit
CAD: Coronary artery disease
SBP: Systolic blood pressure
DBP: Diastolic blood pressure
HR: Heart rate
RR: Respiratory rate
WBC: White blood cells
PLT: Platelet
RDW: Red cell volume distribution width
PT: Prothrombin time
TT: Thrombin time
ALT: Alanine aminotransferase
AST: Aspartate aminotransferase
HCT: Hematocrit
MCV: Mean corpuscular volume
LOS: Length of stay
ICU: Intensive care unit
IQR: Interquartile ranges
CI: Confidential interval
OR: Odds ratio
AUC: Area under the curve
ROC: Receiver-operator characteristic
